# Biomedical Relation Extraction: From Binary to Complex

**DOI:** 10.1155/2014/298473

**Published:** 2014-08-19

**Authors:** Deyu Zhou, Dayou Zhong, Yulan He

**Affiliations:** ^1^School of Computer Science and Engineering, Key Laboratory of Computer Network and Information Integration, Ministry of Education, Southeast University, Nanjing 210096, China; ^2^School of Engineering and Applied Science, Aston University, Birmingham B4 7ET, UK

## Abstract

Biomedical relation extraction aims to uncover high-quality relations from life science literature with high accuracy and efficiency. Early biomedical relation extraction tasks focused on capturing binary relations, such as protein-protein interactions, which are crucial for virtually every process in a living cell. Information about these interactions provides the foundations for new therapeutic approaches. In recent years, more interests have been shifted to the extraction of complex relations such as biomolecular events. While complex relations go beyond binary relations and involve more than two arguments, they might also take another relation as an argument. In the paper, we conduct a thorough survey on the research in biomedical relation extraction. We first present a general framework for biomedical relation extraction and then discuss the approaches proposed for binary and complex relation extraction with focus on the latter since it is a much more difficult task compared to binary relation extraction. Finally, we discuss challenges that we are facing with complex relation extraction and outline possible solutions and future directions.

## 1. Introduction

**Figure 1:**
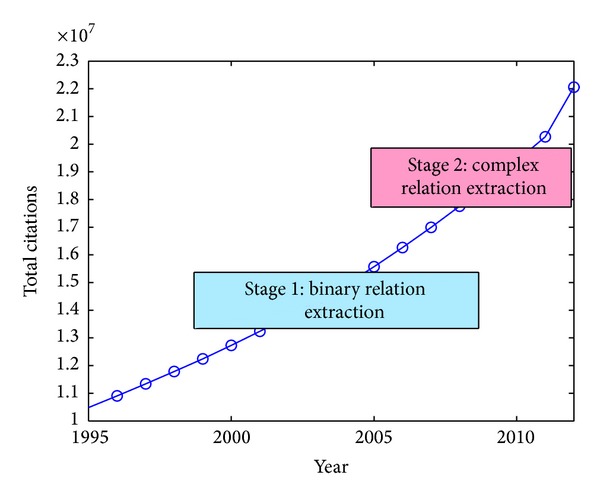
Total bibliographical data in MEDLINE since 1995 and the two stages of biomedical relation extraction research.

To date, more than 22 million bibliographical data such as authors, titles, and abstracts of biomedical articles are available in MEDLINE [1]. These articles reflect the latest development in biomedicine.  Figure 1 shows the growth speed of the total bibliographical data in MEDLINE in recent years. Without assistance, it is hard for scientists or researchers to keep up with the most recent discoveries described in the biomedical literature. Biomedical relation extraction, aiming to automatically discover relations from these biomedical articles with high efficiency and accuracy, is becoming an increasingly well understood alternative to manual knowledge discovery. Its development can be roughly divided into two stages as illustrated in Figure 1.

In the first stage, biomedical relation extraction research worked on the extraction of binary relations such as protein-protein interactions (PPIs), which play a key role in various aspects of the structural and functional organization of the cell. PPIs extraction makes it possible to predict the biological functions of some unknown proteins based on their interacted proteins.  Table 1 shows an example of a sentence with its corresponding PPIs. Early work focused on limited linguistic context and relied on word cooccurrences and pattern matching [2–5]. Later machine learning-based approaches [6–11] were widely employed where extraction models were trained on annotated data enriched with syntactic parsing or semantic parsing results.

However, in reality, complex relations (including *n*-ary relations) are often encountered instead of simple binary relation forms. For example, “…inhibiting tyrosine phosphorylation of STAT6…” describes two biomolecular relations, one is the  phosphorylation  relation, and the other is the complex  negative regulation  relation which is signaled by the word  inhibiting  and takes the first  phosphorylation  relation as its argument. As far as we know, the first paper on complex biomedical relation extraction is the work [12] by McDonald et al. who proposed a framework for extracting variation events from biomedical texts. The variation event, referring to a specific, one-time alteration at the nucleic acid level or amino acid level, was formalized as  variation-type (location, initial-state, and altered-state), an *n*-ary relation with three arguments. After identifying all binary relations between entities, an entity graph was constructed where edges denote the existence of binary relations. The complex relation instances were then constructed by finding the maximal cliques in the graph. After that, extraction of complex biomedical relations such as biomolecular events has attracted much interest. We term it as the second stage of biomedical relation extraction. Several evaluation tasks, such as BioNLP'09 [13], BioNLP'11 [14], and BioNLP'13 [15] shared tasks, have been held in recent years to allow researchers to develop and compare their methods for biomolecular events extraction. Compared to PPIs, biomolecular events describing changes on the state of biomolecules are more complex. Biomolecular event extraction can be used to support the development of biomedical databases.

In this paper, we focus on relation extraction in the biomedical domain, especially complex relation extraction, for which biomolecular event extraction is taken as an example. We present a thorough survey on the methodologies proposed for complex relation extraction. The survey work illustrates the gradual progress of the field and shows the increasing complexity of the proposed methodologies. The rest of the paper is organized as follows. The next section presents a general framework of relation extraction and typical evaluation methods in use. In Section 3, the methods employed in binary relation extraction are summarized. The differences between binary and complex relation extraction are highlighted in Section 4, followed by a survey of methods proposed for complex relation extraction. Finally, challenges in complex relation extraction are discussed and possible solutions and future directions are suggested.

## 2. Relation Extraction in General

In natural language processing, a relation usually refers to a connection between entities in text. There are several types of relations such as semantic relations, grammatical relations, negation, and coreference. Relation extraction here focuses on discovering the semantic relations among several entities [16]. The relation *R* in the relation extraction task can be defined in two possible ways.

*(i) Plain Form. R*≔*r*(*e*_1_; *e*_2_; …; *e*_*n*_), where the *e*_*i*_ is a named entity for *i* = 1,…, *n* and *r* is a predefined relation type (or class).

*(ii) Nested Form. R*≔*r*(*s*_1_; *s*_2_; …; *s*_*n*_), where the *s*_*i*_ is a named entity or a relation defined in plain form or nested form and *r* is a predefined relation type.

When *n* = 1 and *R* is in plain form, *R* is called unary relation. For example, “phosphorylation of STAT6” describes a unary relation  phosphorylation (STAT6). When *n* = 2 and *R* is defined in plain form, *R* is a binary relation. Otherwise, *R* is called complex relation which includes *n*-ary relation. As shown in Table 2, relation_1 and relation_2 are binary and *n*-ary relations, respectively, in plain form, and relation_3 is complex relation in nested form.

**Figure 2:**
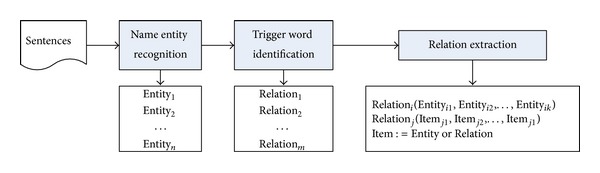
The general framework of a relation extraction system.

Based on the relations defined above, it is straightforward to infer that relation extraction from texts needs to consist of at least three main modules, which are illustrated in Figure 2.

The details of each module are described as follows.

*(i) Named Entity Recognition.* To extract the relations between entities, it is crucial to identify entity names accurately. However, it is not straightforward to precisely identify biomedical entities from texts. One main reason is that entity names are highly polysemous and can refer to completely different entities. It is still a big challenge to normalize entity name mentions. Cohen [17] presented some typical examples of gene name variety and ambiguity. Various methods have been proposed for biomedical entities recognition. In general, these methods can be divided into four categories including dictionary-based, rule-based, machine learning, and hybrid approaches. Experimental results of high recall and precision rates have been reported in [18].

*(ii) Relation Trigger Words Identification.* Relation trigger words identification is similar to the named entity recognition task, but with some differences. The complexity of relation trigger words identification is highly dependent on the relation types. For example, the trigger words for PPIs are usually fixed and can be easily enumerated. A well-designed dictionary is enough for PPI trigger words identification. However, the trigger words for biomolecular events such as positive regulation and transportation are much more difficult. As shown in Table 2, “is sufficient to” is the trigger word for “positive regulation” in this sentence. However, when in a sentence such as “FGF-2 is sufficient to isolate progenitors found in the adult mammalian spinal cord. [PMID: 9417834],” it cannot be treated as a trigger word for positive regulation relations. Therefore, instead of using the simple dictionary based approaches, rule-based and machine learning approaches are widely employed which capture context information as features or patterns.

*(iii) Relation Extraction.* Generally speaking, methods employed in relation extraction module can be roughly classified into two categories, rule-based approaches relying on predefined patterns and machine learning methods based on well-designed features. For rule-based approaches, the predefined patterns may be expressed in forms of regular expressions over words or part-of-speech (POS) tags. Based on these rules, relations between entities that are relevant to specific tasks can be identified. Machine learning-based approaches cast the problem of relation extraction into a classification problem. Suppose to extract the binary relation between *e*_1_ and *e*_2_ in the sentence *S* = *w*_1_ *w*_2_ ⋯ *e*_1_ ⋯ *w*_*j*_ ⋯ *r* ⋯ *e*_2_ ⋯ *w*_*n*_, where *r* is the relation trigger word and the classification function *f* is constructed to output 1 when *e*_1_ and *e*_2_ are related according to relation *r*, otherwise 0. The input to the function *f* is *ϕ*(*S*(*r*, *e*_1_, *e*_2_)), the features extracted from *S*. The function *f* can be constructed as a discriminative classifier such as support vector machines (SVMs). A straightforward way to extend binary relation extraction to *n*-ary relation extraction is to factorize the *n*-ary relation into binary relations and use methods for binary relation extraction. Nevertheless, one issue related to the factorization is that the number of candidate binary relations will grow greatly with the increase of *n*.

To evaluate the performance of a relation extraction system, normally recall and precision values are measured. Suppose a dataset has *P* positive relation instances; a relation extraction system can extract *I* “positive” relation instances. In *I*, only some instances are actually positive which we denote by *TP*. Also the system may falsely extract some relation instances as positive which we denote by *FP*. In *P*, some relation instances are not extracted by the system which we denote by *FN*.

Based on the above definitions, recall and precision can be defined as

(1) $$\begin{array}{c} \text{Precision}=\frac{||TP||}{||TP||+||FP||},\\ \text{Recall}=\frac{||TP||}{||TP||+||FN||}. \end{array}$$

For example, a test dataset has 10 relation instances (||*P*|| = 10). A relations extracting system extracts 11 relation instances (||*I*|| = 11). In *I*, only 6 relation instances (*TP*) can be found in *P*, which are considered as true positive. The remaining 5 relation instances (*FP*) cannot be found in *P*, which are considered as false positive. In *P*, 4 relation instances (*FN*) are not extracted by the system, which are considered as false negative. Thus, the recall of the system is 6/(6 + 4) = 60% and the precision is 6/(6 + 5) ≈ 54.5%.

Obviously, an ideal relation extracting system should fulfil ||*FN*|| → 0, ||*FP*|| → 0. To reflect these two conditions, *F*-score is defined by the harmonic (weighted) average of precision and recall [19] as

(2) Fβ=(1+β2)·Precision·Recall  β2·Precision+Recall  =(1+β2)||TP||(1+β2)||TP||+β2||FN||+||FP||,

where *β* indicates a relative weight of precision.

## 3. Binary Relation Extraction

**Figure 3:**
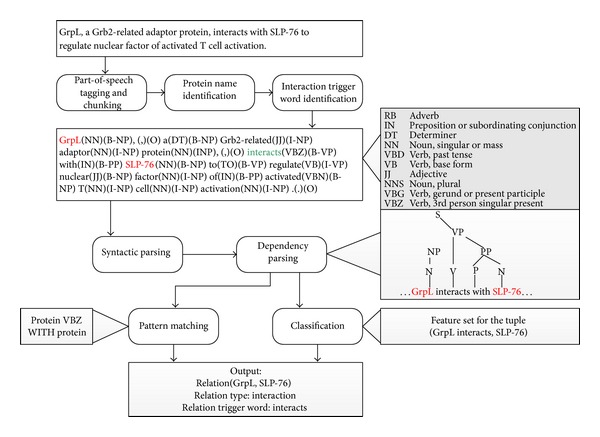
General procedure of a PPI extraction system employing different methodologies.

Substantial amount of work on binary relation extraction in the biomedical domain focuses on extracting PPIs since information about PPIs is crucial for the biologist to uncover the functions of new genes or proteins. In this section, we present an overview of existing techniques for extracting PPIs from the biomedical literature.  Figure 3 illustrates the general procedure of PPI extraction on an example sentence using different approaches. Most PPI extraction systems assume protein names have been normalized and identified. As mentioned before, the words describing interactions between proteins are more likely fixed [20]. Hence, dictionary-based approaches have been widely employed for the detection of the trigger words for PPIs. Methods for PPIs extraction can be broadly classified into two categories, rule-based approaches and machine learning-based approaches, and are described in the following sections. It should be noted that it is not fair to compare the performance of different approaches because different corpora were employed in different approaches.

### 3.1. Rule-Based Methods

In the rule-based approaches, a set of rules [3–5, 10, 21–25] are defined in forms of regular expressions over words or POS tags. Such rules are defined manually or learned automatically from training data. Based on these rules, relations between entities can be recognized.

In [3], gene-gene interactions were extracted using manually constructed linguistic patterns. For example, “gene product acts as a modifier of gene” is a scenario of the predicate  act, which can cover a sentence such as “Eg1 protein acts as a repressor of BicD.”  Egl  and  BicD  can be extracted as arguments of a relation for the predicate  acts. Ono et al. [21] manually defined a set of rules based on syntactic features to process complex sentences, while negation structures were considered as well. An example of the rule in regular expression format is given as  PROTEIN1.∗ not (interact|associate|bind|complex)..∗ PROTEIN2. It achieves good performance with a recall rate of 85% and precision rate of 84% for* Saccharomyces cerevisiae *(yeast) and* Escherichia coli*. Blaschke and Valencia [26] introduced a probability score to each predefined rule depending on its reliability and used it as a clue to score the interactions. Negations and the distance between two protein names were also considered. In [27], PPInterFinder, a web-based text mining tool, was implemented to extract human PPIs from biomedical literature. Firstly, a set of rules were employed to extract PPI candidate pair in the sentences having the abstract forms such as  PROTEIN ∗ RELATION ∗ PROTEIN, RELATION ∗ PROTEIN ∗ PROTEIN,  and  PROTEIN ∗ PROTEIN ∗ RELATION. Then specific syntactic patterns based on the candidate PPI pairs were employed for extracting PPIs. An example of the pattern is given as  S ((NP ≪ PROTEIN1) $++ (VP ≪ RELATION) $++ (NP ≪ PROTEIN2)) (S  denotes sentence,  NP, VP  are POS tags,  ≪  means points to root node, and  $++  means the immediate sisters), which was illustrated in Tregex syntax [28]. Experimental results show that it worked with the accuracy of 66.05% on AIMED corpus and outperformed most of the existing systems.

Manually defined rules require heavy human effort and hence are not easily ported to other domains. It is also not realistic to exhaustedly enumerate rules covering all the possible descriptions of PPIs in text. As such, researchers have resorted to automatically learning PPI extraction rules from data. Phuong et al. [22] used some sample sentences, which were parsed by a link grammar parser, to learn extraction rules automatically. Heuristic rules based on morphological clues and domain specific knowledge were incorporated to remove the negative interactions. Huang et al. [4] employed dynamic programming to learn PPI patterns based on POS tags automatically. Their results gave precision of 80.5% and recall of 80.0%. Liu et al. [24] used PATRICIA trees for learning PPI extraction patterns. All training sentences are inserted and stored in a generic PATRICIA tree. By populating a PATRICIA tree using training sentences, the potential interaction patterns can be extracted. The system achieves an *F*-score of 83.4% in identifying sentences describing interactions between biological entities. In [10], a large set of linguistic patterns was automatically inferred using the information about interacting proteins. Patterns were then refined based on shallow linguistic features and the semantics of dependency types. Experimental results show that a total improvement of 17.2% in *F*-score was achieved on the publicly available PPI corpora.

### 3.2. Machine Learning-Based Methods

Machine learning techniques [6–9, 29–32] were broadly employed for extracting PPIs without human intervention.

Machine learning approaches for PPI extraction typically cast it as a classification problem. A sentence containing a pair of proteins is classified as implying interaction of the pair or not. Under the problem setting, one sentence in the data set yields *C*(*n*, 2) distinct instances, where *n* is the number of different proteins in the sentence and each instance represents a pairwise combination of proteins.

Usually, textual analysis such as POS tagging, syntactic parsing, and dependency parsing is firstly performed on the labeled sentences. A set of selected features can be used for training the classifiers. Apart from that, input to the classifiers can take the form of rich structural representations like parse trees. Based on the nature of the input to the classifier, machine learning-based approaches for relation extraction are further classified into feature-based methods and kernel methods.

For feature-based approaches, syntactic and semantic features are generated from text, serving as cues for deciding whether the entities in a sentence are related or not. Syntactic features used often include two entities, their POS tags, word sequence between them, POS tag sequence between the entities, and syntactic path containing the two entities in the parse tree. Semantic features usually include the path between the two entities in the dependency parse. Based on the complexity of the features employed, feature-based approaches can be further divided into shallow (or partial) parsing based methods and deep (or full) parsing based methods. The former type of methods explores syntactic information which is recovered efficiently and reliably from unrestricted text, by sacrificing completeness and depth of analysis, while the latter type of methods analyzes the entire sentence structure, which normally achieves better performance but with increased computational complexity. In [30], a rich feature set was constructed from multiple parser outputs as shown in Table 3. Firstly bag-of-words, shortest path, and graph features from the output of parsers such as Enju [33] and KSDEP [34] were extracted. According to different feature types and parsers, the output was grouped and features in each group were separately normalized. Then all features from different groups were aggregated into a single feature vector and were subsequently normalized. With feature vectors defined in this way, the system achieved the best performance among all the PPI extraction systems.

The first kernel-based method for PPIs extraction was described in [35] using string-kernels for relation extraction. Given two strings *x* and *y*, the string-kernel computes their similarity based on the number of subsequences that are common to both of them. The more the number of common subsequences, the greater the similarity between the two strings. Other kernels have also been proposed to calculate similarity between the sentences and their syntactic structures, including subsequence kernel [36], tree kernel [37], shortest path kernel [38], graph kernel [39], or a combination of them [40]. Take the graph kernel approach proposed in [39] as an example; a graph kernel was constructed based on the dependency parse of a sentence in biomedical text. Each graph consists of two subgraphs with one describing the dependency structure of the sentence and the other representing the linear order of the words in the sentence. The graph was formalized and represented as an adjacency matrix which was used to get the most likely relation between two proteins.

Supervised machine learning methods have been employed with great success in PPI extraction. However, they usually require a large amount of annotated data for training which are expensive to obtain in practical applications. In [32], unlabeled biomedical texts are employed to enhance the PPI extraction performance using feature coupling generalization. The main idea of feature coupling generalization is to create new features from the cooccurrences of example-distinguishing features and class-distinguishing features in huge unlabeled data. With the generated new features, the system achieved a 60.1% *F*-score and produced significant improvement over supervised baselines.

### 3.3. Available Corpora

Several evaluation tasks have been organized in recent years which help pushing the field of biomedical relation extraction forward. BioCreAtIvE (Critical Assessment of Information Extraction systems in Biology) (http://www.biocreative.org/) began in 2004 and held several times such as BioCreAtIvE I, II, II.5, III, and IV. The key goal of BioCreAtIvE challenge is the active involvement of the text mining user community in the design of the tracks, preparation of corpus, and the testing of interactive systems. The first challenge [41] consists of two common evaluation tasks such as extraction of gene or protein names from text and functional annotation. Later on, the task of extraction of PPIs from text was incorporated in the second challenge [42] in 2007. As an extension of the second challenge, BioCreAtIvE II.5 [43] in 2009 focused on PPIs including ranking articles for curation based on curatable protein-protein interactions and identifying the interacting proteins (using UniProt identifiers) in the positive articles. Following that, the third BioCreAtIvE challenge [44] in 2010 still focused on PPIs and included a gene normalization (GN) task and two protein-protein interaction (PPI) tasks. However, BioCreAtIvE IV [45] held in 2012 paid more focus on curation such as gene ontology (GO) curation and interactive curation.

Genic Interaction Extraction Challenge [46] was associated with learning language in logic workshop (LLL05). The challenge focused on information extraction of gene interactions in* Bacillus subtilin*, a model bacterium. It was reported that the best *F*-score achieved with balanced recall and precision is around 50%.

As annotated corpora are important to the development as well as the evaluation of relation extraction systems, some most notable annotated corpora which are publicly available are listed in Table 4. The first comparative evaluation of the diverse PPI corpora such as AIMed, BioInfer, HPRD50, IEPA, and LLL was presented in [47].

The performance of the representative PPI extraction methods and the data corpora they used are listed in Table 5.

## 4. Complex Relation Extraction

In the molecular biology domain, it is crucial to get detailed views on the behavior of biomolecules. Their behavior is often described in the form of their interplay in molecular events presented in texts. Molecular events describe observable changes of biomolecules, such as binding of proteins or RNA production which can be subdivided into a set of (nested) events. For example, the regulation of gene expression involves at least two events, binding of a transcription factor to a promoter and expression of a protein for a corresponding gene. The descriptions about molecular events spread all over the life science literature. Thus, it is important to extract the nested molecular events, an example of the complex relation from text. Therefore, with the development of biomedical relation extraction, complex relation extraction attracts much more attention with focusing on more specific molecular events, such as gene expression, transcription, protein catabolism, localization and binding, and positive or negative regulation of proteins or other events.

Compared to binary relation extraction, complex relation extraction is a much harder task as elaborated below.

*(i) More Arguments.* While only 2 arguments are involved in binary relations, complex relations may involve more than 2 arguments. Take *n*-ary relation as an example (*n* > 2); it is possible to factorize the *n*-ary relation into *n* − 1 binary relations *r*(*e*_*i*_), *i* = 1,…, *n* and apply the methods described in Section 2 for binary relation extraction directly. Suppose the precision of extracting binary relation is *p*; the precision of extracting *n*-ary relations will be *p* ^*n*−1^ when factorizing the *n*-ary relation into *n* − 1 binary relations. For example, the protein transport event is defined as transport (entity, origin, destination, and location). Assume the precision of binary relation extraction is 0.8; the precision of extracting protein transport event will be 0.8^3^ = 0.512. Hence, directly employing binary relation extraction methods for *n*-ary relation extraction will result in low performance.

*(ii) The Order of Argument List.* Each argument in a *n*-ary relation denotes a specific semantic meaning. Therefore, the order of the arguments is crucial and should be preserved. However, the order of arguments in some binary relations such as PPIs is not important.

*(iii) More Complex Form.* As defined in Section 2, complex relations can appear in two forms, the plain one and the nested one. The nested form is quite common in biomedical events since molecular events are frequently connected by causal relationships and the occurrences of molecular events are closely interconnected. For example, in the text Disruption of curR caused loss of copA expression, the negative regulation of curR leads to a decreased expression of copA, which is described in a nested form with two events as arguments. Hence, the complexity of molecular interactions in organisms requires nesting of molecular events.

*(iv) Ambiguity of Relation Trigger Words.* For binary PPI extraction, the relation trigger words are relatively easy to identify since they are specific to PPIs. Hence, a simple dictionary-based approach can achieve relatively good performance for PPI trigger word detection. On the contrary, trigger words for biomedical events are more difficult to detect. The same word or phrase may or may not refer to a biomedical relation depending on the context. The same word may refer to different relation types in different context. Furthermore, there are many types of biomolecule events compared to PPIs, such as gene expression, transcription, protein catabolism, localization, binding, positive regulation, negative regulation, and phosphorylation.

Apart from the aforementioned four main points, negations and speculations make the complex relation extraction task even more difficult. As such, one subtask of the BioNLP'11 shared task [14] also requires the detection of negation and speculation when evaluating methods for biomedical event extraction.

Most complex relation extraction systems followed the pipeline procedure as the general relation extraction framework described in Section 2, which consists of three modules: term identification, relation trigger word identification, and relation extraction. Few works adopted a nonpipeline approach. For example, Riedel et al. [50] proposed a joint probabilistic model for extracting events based on Markov logic.

Methods for trigger word identification and event extraction are summarized in the following sections. A detailed description of the nonpipeline approaches for complex relation extraction is also presented. Since most research on complex relation extraction in the biomedical domain focused on biomolecular events, we use the term “complex relations” and “biomolecular events” interchangeably in the remainder of this paper.

### 4.1. Event Trigger Word Identification

Event trigger word identification is a key step for biomolecular event extraction. In this section, we categorize approaches for event trigger word identification into three groups: dictionary-based, rule-based, and machine learning-based, and we describe each of them in turn.

#### 4.1.1. Dictionary-Based Methods

Dictionary-based methods differ in the ways of constructing the dictionary [51–58]. Vlachos et al. [52] constructed a dictionary based on the trigger words annotated in the training data. After lemmatizing and stemming, the pairs of trigger stem and event class appearing at least 10 times were kept. In [58], the dictionary was built in three steps: (1) grouping annotated triggers based on their texture values and event types; (2) filtering out triggers belonging to the nontrigger list and triggers that consist of more than two words; the nontrigger list was created from the training data which consists of a list of prepositions and a list of adjectives; triggers were further filtered out by setting a frequency threshold for each event type; (3) calculating a confident score for each trigger based on its frequency being found in the training data. In [51], a dictionary was constructed in the following more elaborated way.

*(i) Step 1.* Collect and lemmatize triggers in the original GENIA event corpus [59] instead of the training data in the BioNLP'09 shared task. 

*(ii) Step 2.* Divide triggers into four groups based on their importance and discrimination. Only those triggers which are important and discriminative or can be disambiguated if not fully discriminative are kept as candidate triggers. 

*(iii) Step 3.* Disambiguate the trigger word *t* belonging to several event types based on the following equation:

(3) $$\begin{array}{c} \text{Imp}\left(t^{T}\right)≔\frac{f\left(t^{T}\right)}{\sum_{s}^{}f\left(s^{T}\right)}, \end{array}$$

where *f*(*t* ^*T*^) refers to the frequency of the trigger *t* with the event type *T* and ∑_*s*_ *f*(*s* ^*T*^) refers to the sum of the frequency of all the triggers with the event type *T*. For the trigger *t*, the event type with the highest Imp will be picked up. For example, consider the trigger word “stimulate” which belongs to two event types: positive_regulation and regulation. Assume that in training data *f*(*t* ^positive_regulation^) = 15, *f*(*t* ^regulation^) = 20, ∑_*s*_ *f*(*s* ^positive_regulation^) = 550, and ∑_*s*_ *f*(*s* ^regulation^) = 500, we can get Ipm(*t*)^positive_regulation^ ≈ 0.0273≺Ipm(*t*)^regulation^ ≈ 0.04. Thus, the regulation relation type is selected for “stimulates.”

#### 4.1.2. Rule-Based Methods

Although dictionary-based methods are quite simple, their performance will be jeopardized when encountering unseen trigger word. Moreover, it is difficult to identify trigger words denoting different relation types in different context. As such, rule-based methods have been employed for trigger word identification [60–62].

**Figure 4:**
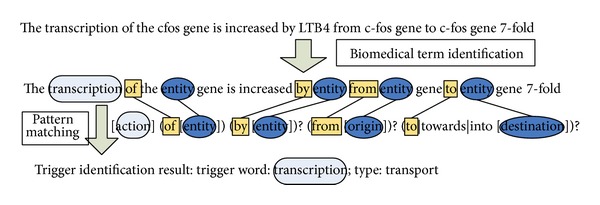
An example of identifying trigger words based on the predefined pattern.

Cohen et al. [61] manually constructed linguistic patterns for each relation type based on the observation of the trigger words in the training data. Each pattern consists of at least one entity argument and one trigger word for a specific relation type, which is written in regular expression. An example of trigger word identification based on the predefined patterns is given in Figure 4 where a manually defined pattern for the protein-transport relation type is employed. It should be mentioned here that to improve the performance of identification, machine learning methods were also employed in [61]. For the sentence  “Leukotriene B4 stimulates c-fos and c-jun gene transcription and AP-1 binding activity in human monocytes,” stimulates, transcription, and binding  might be selected as trigger words based on the predefined rules. After that, a binary classifier was employed to make a judgment using the features of these trigger words. Experimental results show that a combination of rule-based and machine learning-based methods improved the performance of trigger word identification. In [62], heuristic rules were extracted from the training corpus, for instance,  NN/NNS + of + PROTEIN  and  VBN + PROTEIN, to identify candidate triggers. Tokens which are near a protein and have appropriate POS tags were chosen for these rules. For ambiguous trigger classes, the class with the highest occurrence frequency is chosen.

#### 4.1.3. Machine Learning Methods

Both dictionary-based and rule-based methods require manual efforts to construct suitable dictionaries or patterns. Hence, machine learning methods have been explored for trigger words detection [63–70].

It is quite straightforward to apply classification for event trigger word detection. Based on the observation that about 93% of triggers in the training data are single words, given a sentence *S* = *w*_1_, *w*_2_,…, *w*_*n*_, and its annotations, the classification function can be defined as

(4) f(Φ(wi),t) ={+1,If  wi  is  the  trigger  word  with  type  t−1,Otherwise,

where Φ(*w*_*i*_) are features related to *w*_*i*_, which can be extracted from the sentence *S*, and the function *f*(·) decides whether the word *w*_*i*_ is the trigger word with type *t* or not. Most machine learning-based approaches differ in the choice of classification model *f*(·) and feature set Φ(*w*_*i*_).

As mentioned in Section 2, some words or phrases may or may not be the trigger words or may indicate more than one event types depending on the context. For example, “overexpression” can indicate three event types: expression, positive expression, and  negative expression. In [63], multiclass SVMs were utilized for event trigger word identification. A variety type of features was incorporated, such as the token's linear and dependency context and the named entities within the sentence. To alleviate the impact of feature set, two independent SVM classifiers were trained on different feature sets with the same multiclass classification principle. The predictions of the two trigger detectors were combined for final trigger word identification. Lee et al. [64] performed single trigger word detection. To simplify the trigger word identification task, a set of filtering rules was applied to filter out word tokens that are obviously not trigger words, such as “filtering out tokens whose POS tag is not among NN, NNS, and VB,” “filtering out tokens that are a biomedical named entity,” and “filtering out sentences that do not have any proteins.” To train a binary classifier, features derived from words and dependency parse results were used. The word features include the basic word form, POS tags, and the previous word tokens. Features derived from dependency parse results include the dependency path to the nearest protein, whether the word token's child is a proposition, whether the chunk of the child includes a protein, and whether the token's child is a protein and its dependency label is object.

Instead of doing classification on individual words, sequence labeling tries to find the globally best label sequence, which can be used for trigger word identification. Given a sentence *S* = *w*_1_, *w*_2_,…, *w*_*n*_, the sequence labeling approaches find its label *Y* = *y*_1_, *y*_2_,…, *y*_*n*_, where each *y*_*i*_ ∈ *T* and *T* is a set of event types. This is a hard problem since the number of possible *y*_1:*n*_ is too high. Additional assumptions on output labels can be made to solve the problem. MacKinlay et al. [71] presented an approach based on conditional random fields (CRFs). CRFs, a single exponential model for the joint probability of an entire sequence of labels given an observation sequence, can model sequential effects and support the use of a large number of features. Feature types such as word-forms, lemmas, POS, chunk tags, protein annotation, and grammatical dependencies were employed. To further improve the performance of trigger identification, a simple dictionary of trigger words was constructed from the training data. For a given term, the occurrence frequency of its associated event classes in the training data was calculated and the one with the highest occurrence frequency was selected. The result was combined with the output of CRFs to generate a final trigger word list.

### 4.2. Event Extraction

Given a set of candidate trigger words, we need to associate them with appropriate arguments for event extraction. There are several different methods aimed at argument detection [52, 54, 58, 61, 62, 72–78]. These approaches again can be classified as either rule-based or machine learning-based as mentioned in Section 2. Machine learning approaches are further divided into feature-based or kernel-based ones.

#### 4.2.1. Rule-Based Approaches

Rule-based methods rely on a set of manually defined or automatically generated rules for biomedical event extraction [52, 54, 58, 61, 62, 72, 73, 76, 79]. The rules are usually expressed as regular expressions over words or POS tags.

In [62], patterns were constructed from dependency parse results using the following steps.

*(i) Parsing and Pruning.* The parse tree for each sentence containing at least one trigger word was generated and pruned by removing nodes which contain only one child and that child node has zero or one descendant. 

*(ii) Identification.* Candidate arguments of events were identified by combining entity and event trigger in a sentence. At least one trigger with one protein or event is involved in the combination. The number of argument is usually less than 5. 

*(iii) Pattern Extraction.* After identification, concepts of arguments in each combination were assigned to parse tree nodes based on the span of argument and content of nodes. Patterns were extracted from the parse trees and some were discarded if the parse trees they cover have the depth exceeding a predefined threshold.

**Figure 5:**
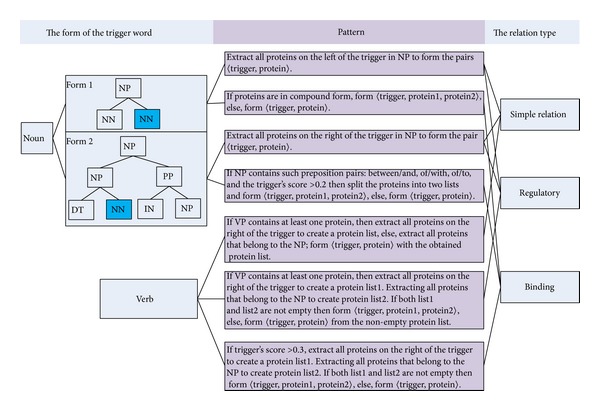
Event extraction rules employed in [58].

Bui and Sloot [58] defined rules based on the syntactic patterns involving trigger words as shown in Figure 5. These rules were defined based on the POS tag and event type of the trigger word. For the trigger words in noun form, a joint node of the trigger word and one or more proteins from the parse trees were examined to form a possible biomedical event. For the trigger word in verb form, the direct parent of the trigger word and a sister NP adjacent with the trigger word are extracted to form a possible biomedical event. For those trigger words which are adjective and are compound such as  protein-mediated, the same rules are applied to extract an event as ones for trigger words in noun form. Otherwise the rules used for trigger words in verb form are applied.

Instead of constructing patterns manually, Liu et al. [79] built the biological event rules automatically from training sentences. At first, the directed dependency graph of each training sentence was transformed into an undirected one by dropping the direction of each edge. Then for each event in the training data, the shortest dependency path in the undirected graph connecting the event trigger nodes to each event argument node was selected. All shortest dependency paths corresponding to an event were unified. The original directed dependency representation of the unified path was retrieved which is considered as an extraction pattern for that event. Given a sentence together with its dependency parse graph, the event extraction task is transformed to a subgraph matching problem which aims to search for a subgraph isomorphic to graph patterns corresponding to certain events.

#### 4.2.2. Feature-Based Approaches

**Figure 6:**
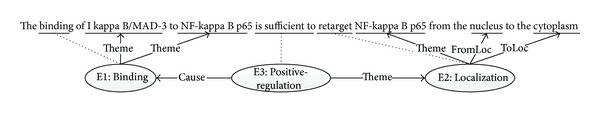
An example of a sentence with target event edge to be extracted.

As shown in Figure 6, a sentence and its corresponding event information are illustrated in structure edge. The argument detection task can be transformed into the edge detection problem. Similar to binary relation extraction, machine learning methods for complex relation extraction can also be categorized into feature-based or kernel-based methods. It should be noted that most machine learning approaches for event extraction cast the *n*-ary relation extraction into several binary relation extractions.

Features-based approaches rely on elaborately selected features to construct classifiers [55, 63, 71, 73, 80–83]. Generally, syntactic and semantic features are extracted from the text to construct a classifier. Syntactic features include the word level feature of the entities such as base form, POS tag, and path in the parse tree, while semantic features are usually extracted from the path in the dependency parse.

The best performing system [63] in the BioNLP'09 shared task chose a wide range of features for multiclass SVM classifier training. The features employed are listed in Table 6. Experimental results showed that the performance of the system was heavily dependent on the features extracted from dependency parse results. In particular, it was noted that the shortest undirected path of syntactic dependencies in the Stanford scheme parse of a sentence accurately captures the relationship expressed among arguments involved in the biomedical events. McGrath et al. [83] presented a signature-based machine learning method for biomedical event extraction. Inspired by features used in semantic role labeling, the feature set used for classification includes the type of event trigger words, argument terms, argument type, parse tree path, and so forth. More details of the features used can be found in Table 6. Each pair of trigger word and event argument act as an instance for SVM classifier training. Experimental results showed that among all the employed features, the most discriminative features were argument terms and argument type. In [73], features such as the neighboring words, their POS tags of the trigger words or proteins, and the distance between the left and right proteins of the trigger words were used to train a classifier for the identification of event arguments. Each pair of trigger word and protein was classified into one of the nine events (the keyword as an event trigger and the protein name as the theme of the event) and two nonevent classes (nonevent, keyword not an event trigger or  wrong-protein; the theme of the event is a different protein). In [55], the feature set was extended from the one used in PPIs extraction. Instead of extracting binary relations and only one path in the dependency graph, more complex subgraphs were processed and trigrams were included in the feature set.

#### 4.2.3. Kernel-Based Approaches

**Figure 7:**
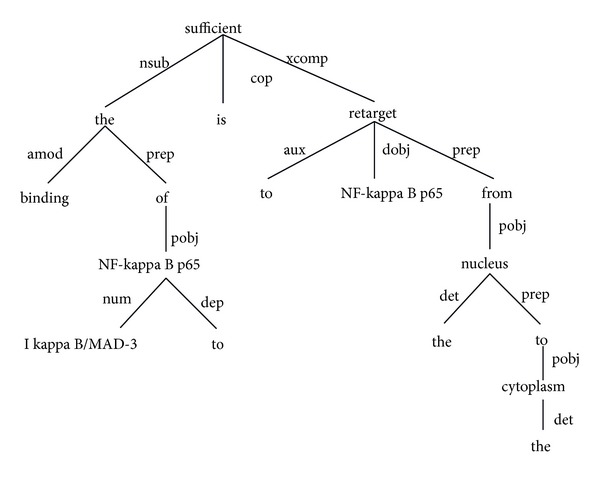
The dependency path for the sentence “The binding of I kappa B/MAD-3 to NF-kappa B p65 is sufficient to retarget NF-kappa B p65 from the nucleus to the cytoplasm.”

To remedy the problem of selecting a suitable feature-set, specialized kernels are designed to exploit the rich representations of the input data like shallow parse trees. The kernel-based approaches concern about the design of proper kernels for similarity measurement between two sentences [51, 84]. A kernel function is typically defined in [84]:

(5) $$\begin{array}{c} K\left(x,y\right)=e^{-\gamma (\text{edit\_distance}(x,y))}, \end{array}$$

where *x*, *y* are the two dependency relation paths and edit_distance (*x*, *y*) is the word-based edit distance between *x*, *y*. The dependency path for the sentence in Table 2 is illustrated in Figure 7. Given the two dependency paths “PROTEIN - nsubj - interacts - prep with - PROTEIN” and “PROTEIN - nsubj - interacts - prep with - PROTEIN - conj and - PROTEIN,” the edit distance between two paths is 2 since the first path can be converted into the second one by inserting “PROTEIN” and “conj and.” The edit distance is normalized to take values in the range between 0 and 1 by dividing it by the length (number of words) of the longer path. Each candidate trigger and argument pair are classified and the SVM score is used to disambiguate the event types, if a candidate trigger matches a trigger in more than one of the event classes. A trigger which is ambiguous among the event types in the same class is assigned to the event type for which it is most frequently used as a trigger. The overall performance of the kernel-based system was 30.42% recall, 14.11% precision, and 19.28% *F*-score on the BioNLP'09 shared task. Feature-based approach was also employed in [84]. Both achieved similar performance.

The kernel employed in [51] is a converted form of dependency graph in which each dependency node was represented by a set of labels associated with that node. The dependency edges were also represented as nodes in the new graph. The entire graph was represented as an adjacency matrix. It was further processed to contain the summed weights of paths connecting two nodes of the graph. Irrelevant lexical information was trimmed and pruned from a dependency graph. Afterwards, abstract conceptual class information such as protein name was added into the graph. Experimental results showed that it achieved 45.8% precision, 47.5% recall, and 46.7% *F*1-score and scored second in the BioNLP'09 shared task.

To summarize, we have seen technology advancement in biomedical event extraction. However, the performance of biomedical event extraction systems highly depends on the coverage and size of the training data. Without enough training data, the performance of the system based on machine learning approaches will be jeopardized. One possible solution is to employ multiple corpora to achieve broad semantic coverage and high accuracy. In [86], to learn from multiple corpora with partial semantic annotation overlaps, a filtering approach was proposed. Based on the filtering approach, a new partially overlapping corpus was added with the benefit of increasing both the positive examples of overlapping semantic types, as well as increasing the set of negative instances of these types. Experimental results showed that learning from overlapping corpora can produce a single, corpus-independent, wide coverage extraction system that outperforms systems trained on a single corpus.

### 4.3. Methods Based on Joint Model

As mentioned in Section 2, most relation extraction systems follow a pipeline architecture where each module is a simple task-specific local classifier. One drawback of such a pipeline architecture is that errors introduced in early stages of a pipeline will be cascaded to the later stages. As such, recent research has started to investigate a joint discriminative model for relation extraction [85, 87].

To employ joint model for relation extraction, it is crucial to represent the events in a sentence through a set of binary variables. After that, the search for event structures can be easily formulated into an optimization problem. In [85], the representation is conducted as follows. Given a sentence *S* = (*w*_1_, *w*_2_,…, *w*_*n*_), each word *w*_*i*_, *i* = 1,…, *n* is labeled with the event type if it is a trigger, or  None  if it is not a trigger. This labeling is represented through a set of binary variables *e*_*i*,*t*_, one for each possible event type *t*. For each candidate trigger *w*_*i*_, the arguments of all events that have *w*_*i*_ as trigger are considered. An edge *w*_*i*_ → *w*_*j*_ denote *w*_*j*_ is an argument of the event with *w*_*i*_ as trigger. The edge is represented through a binary variable *a*_*i*,*j*,*r*_, where *r* ∈ *R* is the argument role such as  Theme,  Cause,  and None. Furthermore, pairs of proteins that are themes in the same binding event are represented with an edge. For two protein tokens *p* and *q* this edge is represented through the binary variable *b*_*p*,*q*_. An example of a sentence and its corresponding representation is given in Table 7. The event annotation can be found in Table 2.

Based on the representation described above, three models were proposed in [85]. The first model performs a simple way of joint trigger and argument extraction. It independently scores trigger labels and argument roles based on the following equation:

(6) $$\begin{array}{c} S_{1}=\underset{e_{i,t}=1}{\sum }S_{T}\left(i,t\right)+\underset{a_{i,j,r}=1}{\sum }S_{R}\left(i,j,r\right), \end{array}$$

where *S*_*T*_(*i*, *t*) is the score function measuring how well the event label *t* fits to token *w*_*i*_ and *S*_*R*_(*i*, *j*, *r*) is the score function measuring the compatibility of role *r* as label for the edge *w*_*i*_ → *w*_*j*_. Using the scoring function from the first model, the second model enforces additional constraints that ensure consistency between events in hierarchical regulation structures. For example, every active edge must either end at a protein or at an active event trigger. The third model includes the first two and explicitly captures which arguments are part of the same event. The scoring function for the third model is

(7) $$\begin{array}{c} S_{3}=S_{2}+\underset{b_{p,q}=1}{\sum }S_{B}\left(p,q\right), \end{array}$$

where *S*_*B*_(*p*, *q*) is the protein-pair score function based on a feature representation of the lexical and syntactic relation between two proteins. Introducing a binding variable *b*_*p*,*q*_ into the scoring function enforces an additional constraint that the same pair of entities *p*, *q* cannot be arguments in more than one event. When evaluated on the BioNLP 2009 shared task, the first two models outperform the previous best joint approach and are competitive when compared to the existing best performing model. The third model achieves the state-of-the-art result.

### 4.4. Available Corpora

The BioNLP'09 shared task [13] concerns about the recognition of biomolecular events that appear in biomedical literature. The shared task consists of three subtasks:* core event extraction*,* event enrichment*, and* negation and speculation recognition*. The three subtasks are illustrated in Table 8 with three example sentences where their event information corresponds to the three subtasks.* Core event extraction*, as shown in the first row of Table 8, includes trigger detection (Expression), event typing (Gene_expression:Expression), primary argument recognition (IkappaBalpha), and finally frame filling (E1 event_type:event trigger Theme:primary argument). For* event enrichment*, the secondary arguments are found and added to the event frame as  ToLoc: nuclear  as shown in the second row of Table 8. For* negation and speculation recognition*, negations and speculations of events need to be identified and formatted as  M1 Negation/Speculation E1  where E1 denotes the event information recognized in the* core event extraction* and* event enrichment* subtasks.

The BioNLP'11 shared task [14] is the follow-up event of the BioNLP'09 shared task. It extended from the BioNLP'09 shared task in three aspects: text type, domain, and targeted event types. Event extraction tasks are arranged in four tracks, GENIA, epigenetics and posttranslational modifications (EPI), infectious diseases, and bacteria. The GENIA task aims at extracting events occurring on genes or gene products, the same as BioNLP'09 shared task. The corpus for GENIA task consists of texts drawn from abstracts and full texts in the transcription factors in human blood cells domain, annotated for nine event types involving proteins. The EPI task focuses on events relating to epigenetic change, including DNA methylation and histone modification, as well as other common posttranslational protein modifications. The corpus for the EPI track consists of abstracts relating primarily to protein modifications, drawn from MEDLINE without other subdomain restrictions and annotated for 14 protein entity modification event types and their catalysis.

Moreover, to encompass different biological levels from molecule to organism, the multilevel event extraction (MLEE) corpus [88] consists of abstracts in the blood vessel development subdomain annotated using a comprehensive set of entity and event types.

The performance of all the submitted biomedical event extraction systems of BioNLP'09 and BioNLP'11 can be found in [13, 14]. The best overall performance on GENIA (56.04%, *F*-score) in BioNLP'11 was achieved by [89], demonstrating a significant improvement when compared to the best performance in BioNLP'09 (51.95%, *F*-score) achieved by [63]. For other biomedical event extraction systems that did not participate in the shared tasks, their performance results are listed in Table 9.

## 5. Challenges and Future Directions

The continuing growth and diversification of the scientific literature, a prime resource for accessing worldwide scientific knowledge, will require tremendous systematic and automated efforts to utilize the underlying information. In molecular biology, molecular events describe observable changes of biomolecules, such as binding of proteins or RNA production. In parallel to molecular formations, these molecular events influence the formation of a phenotype, which may be responsible for drug reactions or development of certain diseases. As such, biomolecular event extraction attracted much research interests recently. We have witnessed the advancement of technologies developed for biomedical event extraction, ranging from simple rule-based pattern matcher to sophisticated, hybrid parser employing computational linguistics methods and machine learning. Nevertheless, biomedical event extraction still faces the following significant challenges.

*(i) High Complexity of Molecular Events.* In molecular events, all biological processes can be subdivided into a set of molecular processes which are nested and interconnected. For example, regulation of a gene expression involves many subprocesses, such as binding of a transcription factor to a promoter, activation of a promoter of a corresponding gene or even operon for gene transcription, transcription of DNA snippets into RNA structures, and translation of RNA structures into proteins. Thus, molecular events are very complex. To obtain a comprehensive view of one molecular event, we need to know all the other molecular events which are associated with it and all events which might cause this particular event. To capture an overall picture of biomedical events, different levels of biological organization from the subcellular to the organism level should be considered, instead of binding and phosphorylation events which are only part of biological systems.

One possible way to deal with the complexity of biomedical events is to explore the use of ontologies which can provide the basic hierarchical information for biomolecular events. Ontologies, structured lists of terms, are often used by natural language processing (NLP) technologies to establish the semantic function of a word in a document. A popular ontology in biomedicine is gene ontology (GO) [90]. To the best of our knowledge, none of the existing biomedical event extraction systems make use of information from ontologies. In [91], an ontology-based system was constructed to detect different types of business events from unstructured sources of information for business documents analysis. The system achieved 95% precision and 67% recall in detecting all supported business event types from newspaper texts. We speculate that combining information extraction with ontologies could abstract away from details of complex relations and will potentially make the detection of complex relations easier. 

*(ii) Relatively Low Performance of Existing Systems.* As shown in Section 4, the best method for biomedical event extraction evaluated on the BioNLP'11 shared task gives an *F*-score of about 0.57, which is still relatively low. While this shows that the task itself is very difficult, another possible reason is the lack of annotated training data. However, it is quite expensive and time-consuming to manually annotate training data. Therefore, semisupervised learning approaches, which employ both unlabeled and labeled data, should be considered as an alternative solution. Method of automatically enlarging annotated corpora based on distant supervision has already shown some success [92]. Another possible solution is using active learning instead of randomly selecting sentences for annotation since active learning only chooses training examples that are most useful for learning. Active learning has already demonstrated its effectiveness for speeding up the creation of semantically (named entity and relationship) annotated corpora in different language domains, including the biomedical field [93]. An interactive annotation process involving end users can support more rapid creation of annotated corpora in the biomedical domain. 

*(iii) Unsolved Problems in NLP.* Some problems exist not only in the field of biomedical event extraction, but also in the general field of NLP. Two of them are (1) dealing with negative sentences, which is considered as a well-known problem in language understanding [94]; a pattern-based approach [95] for negation recognition achieved an accuracy of 0.943 on clinic texts, which might provide some clues for solution; (2) resolving coreferences, the recognition of implicit information in some sentences may contain key information, for example, protein names and events, that are later mentioned in other sentences. 

*(iv) Gap Between Biologists and Computational Scientists.* Bridging the gap between biologists and computational scientists seems to be crucial to the success of biomedical event extraction. Currently, this field is dominated by researchers with computational background. However, the biomedical knowledge is only possessed by biologists. That is crucial for defining standards for evaluation, for identification of specific requirements, potential applications, and integrated information system for querying, visualization, and analysis of data on a large scale, and for experimental verification to facilitate the understanding of biological interactions. Hence, to attract more biologists into the field, it is important to design simple and friendly user interfaces that make the tools accessible to nonspecialists.

## Figures and Tables

**Table 1:** Example of a sentence and its corresponding PPIs.

Sentence	Leukotriene B4 stimulates c-fos and c-jun gene transcription and AP-1 binding activity in human monocytes.
PPIs	Stimulate (leukotriene B4, c-fos)
	Stimulate (leukotriene B4, c-jun)
	Stimulate (leukotriene B4, AP-1)

**Table 2:** Example of a sentence and the relations it contains.

Sentence	The binding of I kappa B/MAD-3 to NF-kappa B p65 is sufficient to retarget NF-kappa B p65 from the nucleus to the cytoplasm.
Relation_1	Binding (I kappa B/MAD-3, NF-kappa B p65)
Relation_2	Localization (NF-kappa B p65, the nucleus, and the cytoplasm)
Relation_3	Positive regulation (relation_1, relation_2)

**Table 3:** Features employed in [30].

Feature type	Features in detail
Word level	Lemma form of a word; relative position to the pair of proteins (before, middle, after); frequency in the sentence

Shortest path level	Vertex walks in the shortest path; edge walks in the shortest path; subsets of walks on the target pair in a parse structure

Graph level	Graph matrices based on a parse structure subgraph and linear order subgraph from the dependency parsers. The graph features are all the nonzero elements in the graph matrices

**Table 4:** Available annotated corpora for binary relation extraction in the biomedical domain.

Corpus name	General description	URL
GENIA	2,000 MEDLINE abstracts with more than 400,000 words and almost 100,000 annotations for biological terms.	http://www.nactem.ac.uk/genia/genia-corpus

LLL05	80 sentences in the training set including 106 examples of genic interactions without coreferences and 165 examples of interactions with coreferences.	http://genome.jouy.inra.fr/texte/LLLchallenge/

BioCreAtIvE II	Training data is derived from the content of the IntAct and MINT databases. The test set collection consists of a collection of PubMed article abstracts.	http://www.biocreative.org

AIMed	225 MEDLINE abstracts (200 abstracts describing interactions between human proteins and around 1000 tagged interactions).	ftp://ftp.cs.utexas.edu/pub/mooney/bio-data

BioInfer	1100 sentences annotated with protein names, their relationships, and PPI annotations.	http://mars.cs.utu.fi/BioInfer/

HPRD50	50 abstracts referenced by the Human Protein Reference Database including 266 relation instances.	http://www.hprd.org

**Table 5:** Performance of existing PPI extraction methods on the data corpora used.

Category	Result (%)		Corpus	References
	Recall	Precision		
Rule-based	86.8	94.3	834 and 752 sentences obtained by a MEDLINE search using these keywords, “protein binding,” “yeast,” “*E. coli*,” “protein,” and “interaction.”	[21]
	60	87	550 sentences were retained containing at least one of four keywords “interact,” “bind,” “associate,” “complex,” or one of their inflections from 3343 abstracts retrieved from MEDLINE with the following keywords: “*Saccharomyces cerevisiae*,” “protein,” and “interaction.”	[22]
	80.0	80.5	About 1200 sentences were kept from the top 50 biomedical papers retrieved from the Internet by querying using the keyword “protein-protein interaction.”	[4]

ML methods	57	90	Training set consists of 500 abstracts from MEDLINE. Evaluation set consists of 56 abstracts collected using search strings “protein” and “inhibit.”	[48]
	21	91	3.4 million sentences from approximately 3.5 million MEDLINE abstracts dated after 1988 containing at least one notation of a human protein.	[49]
	71.9	60	AIMed	[38]
	87.2	72.5	LLL	[39]
	76	70	The test corpus consists of 300 randomly selected sentences.	[24]
	70.7	70.3	LLL	[10]
	71.9	60	AIMed	[30]
	59.26	63.37	LLL	[9]
	89	73	LLL	[11]

**Table 6:** Features sets and classifiers employed in machine learning-based approaches for event extraction.

References	Feature sets	Classifier
[63]	(1) N-grams (merging the attributes of 2 to 4 consecutive tokens); (2) individual component features for each token and edge in a path; (3) semantic node features (the attributes of the two terminal event/entity nodes of the potential event argument edge); (4) frequency features (the length of the shortest path and the number of named entities and event nodes, per type, in the sentence)	Multiclass SVM

[83]	(1) Trigger type; (2) argument terms; (3) argument type; (4) argument supertype; (5) trigger and argument; (6) trigger and argument POS; (7) parse tree path; (8) voice of sentence (active or passive); (9) trigger and argument partial paths; (10) trigger subcategorization	SVM

[73]	(1) Words and POS in a window around the trigger; (2) distances between the trigger and the two nearest annotated proteins (left and right) and the theme candidate	C4.5 decision tree

[55]	(1) Three stemmed consecutive words from the subsentence spanning the event; (2) lexical and syntactic information of triggers; (3) size of the subgraph; (4) bag of words; (5) length of the subsentence; (6) extra features for regulation events; (7) vertex walks which consist of two vertices and their connecting edge	SVM

**Table 7:** An example of a sentence and its event representations employed in [85].

Sentence	$\frac{\text{The}}{1}\frac{\text{binding}}{2}\frac{\text{of}}{3}\frac{\text{I kappa B/MAD-3}}{4}\frac{\text{to}}{5}\frac{\text{NF-kappa B p65}}{6}\frac{\text{is}}{7}\frac{\text{sufficient}}{8}\frac{\text{to}}{9}\frac{\text{retarget}}{10}\frac{\text{NF-kappaBp65}}{11}\frac{\text{from}}{12}\frac{\text{the}}{13}$ $\frac{\text{nucleus}}{14}\frac{\text{to}}{15}\frac{\text{the}}{16}\frac{\text{cytoplasm}}{17}$.
Event representations	*e*_2,Binding_ = 1, *e*_10,Localization_ = 1, *e*_8,Positive-regulation_ = 1
	*a*_2,4,Theme_ = 1, *a*_2,6,Theme_ = 1
	*a*_10,11,Theme_ = 1, *a*_10,14,FromLoc_ = 1, *a*_10,17,ToLoc_ = 1
	*a*_8,2,Cause_ = 1, *a*_8,10,Theme_ = 1
	*b*_4,6_ = 1

**Table 8:** Examples of the three subtasks of the BioNLP'09 shared task.

Subtask	Sentence	Events
Core event extraction	*Expression of IkappaBalpha* in the nucleus of human peripheral blood T lymphocytes.	**E1 Gene_expression**: Expression **Theme**: IkappaBalpha

Event enrichment	We demonstrate the *nuclear localization of I(kappa)B(alpha)* in PBL by different techniques: Western blot, indirect immunofluorescence, and electron microscopy.	**E1 Localization**: localization **Theme**: I(kappa)B(alpha) **ToLoc**: nuclear

Negation and speculation recognition	This *failure to degrade IkappaBalpha* may underlie both the observed decrease in NFkappaB induction and the IL-2 receptor expression in TNF-treated T cells during aging.	**E1 Protein_catabolism**: degrade **Theme**: IkappaBalpha **M1 Negation** E1

**Table 9:** Performance of the biomedical event extraction systems not participating in the BioNLP shared tasks.

Category	Recall (%)	Precision (%)	*F*-score (%)	Corpus	References
Nonpipeline	NA	NA	56.0	BioNLP'11	[87]
	NA	NA	**57.4**	BioNLP'11	[85]

Rule-based	38.01	52.06	43.94	BioNLP'11	[58]
	33.66	41.77	37.28	BioNLP'09	[79]
	10.12	27.17	14.75	BioNLP'11	[62]

Machine learning	51.25	64.92	57.28	BioNLP'11	[86]
	NA	NA	**53.15**	BioNLP'09	[82]
	NA	NA	53.30	BioNLP'11	[82]
